# Differential kinetics of the cardiac, ventilatory, and gas exchange variables during walking under moderate hypoxia

**DOI:** 10.1371/journal.pone.0200186

**Published:** 2018-07-25

**Authors:** Naoyuki Ebine, Tomoko Aoki, Masahiro Itoh, Yoshiyuki Fukuoka

**Affiliations:** 1 Faculty of Health and Sports Sciences, Doshisha University, Tatara, Kyotanabe, Kyoto, Japan; 2 Faculty of Environmental and Symbiotic Sciences, Prefectural University of Kumamoto, Higashiku, Kumamoto, Japan; 3 Department of Physiology, Kumamoto University Graduate School of Life Sciences, Chyoku, Kumamoto, Japan; Tokai University, JAPAN

## Abstract

We investigated the effects of moderate hypoxia (FiO_2_ = 15%) on different kinetics between pulmonary ventilation (${\dot{V}}_{E}$) and heart rate (HR) during treadmill walking. *Breath-by-breath* ${\dot{V}}_{E}$, oxygen uptake ($\dot{V}O_{2}$), carbon dioxide output ($\dot{V}CO_{2}$), and HR were measured in 13 healthy young adults. The treadmill speed was sinusoidally changed from 3 to 6 km·h^-1^ with four oscillation periods of 1, 2, 5, and 10 min. The amplitude (*Amp*), phase shift (*PS*) and mean values of these kinetics were obtained by harmonic analysis. The mean values of all of these responses during walking at a sinusoidally changing speed became greater under hypoxia compared to normoxia (FiO_2_ = 21%), indicating that moderate hypoxia could achieve an increased energy expenditure (increased $\dot{V}O_{2}$ and $\dot{V}CO_{2}$) and hyperventilation. The *Amp* values of the ${\dot{V}}_{E}$, $\dot{V}O_{2}$, and $\dot{V}CO_{2}$ kinetics were not significantly different between normoxia and hypoxia at most periods, although a significantly smaller *Amp* of the HR was observed at faster oscillation periods (1 or 2 min).The *PS* of the HR was significantly greater under hypoxia than normoxia at the 2, 5, and 10 min periods, whereas the *PS* of the ${\dot{V}}_{E}$, $\dot{V}O_{2}$, and $\dot{V}CO_{2}$ responses was not significantly different between normoxia and hypoxia at any period. These findings suggest that the lesser changes in *Amp* and *PS* in ventilatory and gas exchange kinetics during walking at a sinusoidally changing speed were remarkably different from a deceleration in HR kinetics under moderate hypoxia.

## Introduction

Hiking is a very popular pastime in Japan—in fact, approx. "300,000 people have hiked to the top of Mount Fuji alone. Modeling the experience of mountain hiking in an experimental setting can be difficult, since mountain landscapes are rugged and the atmosphere is often hypoxic. In this study, in an attempt to accurately model the physiological cardiovascular responses of walking along mountain paths, we used treadmill walking in combination with the sinusoidal oscillation of walking speed and changes in the inspiratory oxygen concentrations (FiO_2_).

We also used sinusoidal work loading to more precisely define the kinetic response characteristics [1,2]. Dynamic physiological responsiveness can be estimated by the phase shift (*PS*) as the time lag and the amplitude (*Amp*) of the heart rate (HR) ventilatory and gas exchange variables in response to sinusoidal exercise [3–6], which can provide a clear and simple evaluation of an individual’s HR and ventilatory and gas exchange responses.

It has been well established that hypoxia speeds up the kinetics of CO_2_ output ($\dot{V}CO_{2}$) and ventilation (${\dot{V}}_{E}$) via the peripheral chemoreceptors (i.e., carotid bodies) [7–9]. On the other hand, hypoxia also slows the kinetics of O_2_ uptake ($\dot{V}O_{2}$) [7,9–13] and HR [7,9,10, 12,13] at the onset of step exercise. The HR and cardiac output ($\dot{Q}$) values during steady-state exercise are significantly increased under hypoxia compared to normoxia [13]. Thus, hypoxia can change the blood flow into exercising muscle [14] and slow the kinetics of cardiovascular responses during exercise in order to compensate for the utilization of O_2_ in working muscles. Consequently, the $\dot{V}O_{2}$ values are similar between normoxia and hypoxia during steady-state exercise. It was reported that these observations were supported only by steady-state exercise [13,15]. However, severe hypoxia and/or non-steady-state exercise mode (e.g., gradual load variation) would be able to achieve greater energy expenditure (i.e., an increased $\dot{V}O_{2}$ and $\dot{V}CO_{2}$) even at the same absolute work intensity [15].

In the present study, our goal was to quantify the effects of acute moderate hypoxia on the kinetics of cardiac, ventilatory and gas exchange variables during non-steady-state walking. We hypothesized that inherent hypoxia would decelerate the HR kinetics, whereas it would accelerate the ${\dot{V}}_{E}$ kinetics during sinusoidal walking as a model of mountain hiking. To test this hypothesis, we compared changes in the kinetics of HR, ${\dot{V}}_{E}$ and gas exchange in response to sinusoidal changes in walking speed between normoxic and moderately hypoxic states.

## Subjects and methods

### Subjects

Thirteen healthy young volunteers (seven males and six females) with no cardiac, ventilatory, gas exchange or musculoskeletal disorders participated. The mean age, height, and body weight values were 20.5 ± 1.6 years old, 1.66 ± 0.08 m, and 58.0 ± 7.3 kg, respectively (mean ± standard deviation [SD]). Written informed consent was obtained from all subjects after a detailed explanation of all procedures, the purpose of the study, the possible risks, and the benefits of participation. Our study conformed to the Declaration of Helsinki, and the ethical committees of the Prefectural University of Kumamoto and Doshisha University approved all study procedures.

### Experimental protocols

This study was carried out on a motor-driven treadmill (modified TMS 2200; Nihon Kohden, Tokyo). All 13 subjects performed a series of submaximal exercise tests as described [16]. The inspired oxygen fraction (FiO_2_; %) was set at either normobaric normoxia (20.9% FiO_2_) or moderate normobaric hypoxia (15.0% FiO_2_) [17–19]. Hypoxic gas was supplied via a 1000-L Douglas bag with a hypoxic gas generator system (Everest Summit II; Will Co., Tokyo). The atmosphere in the normobaric environmental chamber was set at 25°C with a relative humidity of 50%.

**Fig 1:**
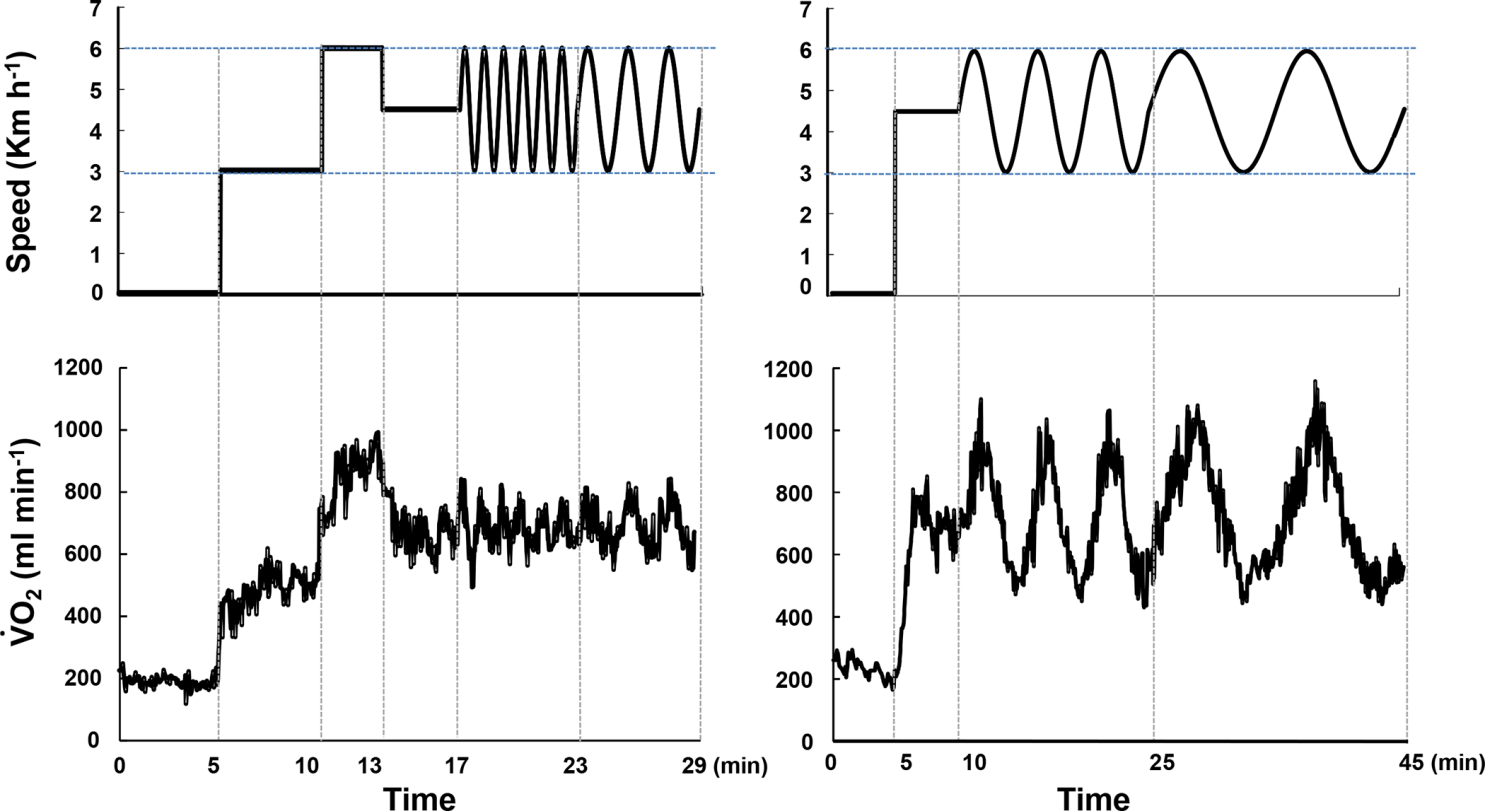
Walking protocol at sinusoidally changing speed following three different constant walking speeds. Treadmill walking was started after 5 min of rest, and consisted of 5 min at 3 km·h^−1^, 3 min at 6 km·h^−1^, and 4 min at 4.5 km·h^−1^ for a total of 12 min of walking. After three consecutive exercises, the sinusoidal speed change between 3 and 6 km·h^−1^ was controlled by a PC in four different periods (1, 2, 5, and 10 min) (upper panel). At the same time, the measured *breath-by-breath* $\dot{V}O_{2}$ values were obtained (lower panel).

Following a 4 min warm-up by the subject consisting of walking at a constant speed at the midpoint of the sinusoid (4.5 km·h^-1^), we changed the treadmill speed with a sinusoidal pattern from 3 km·h^-1^ to 6 km·h^-1^ at a periods (*T*) of 10, 5, 2 or 1 min (Fig 1). Before the sinusoidal pattern, a stepwise manner (steady-state) pattern at speeds of 3 km·h^-1^ and 6 km·h^-1^ for 5 min and 3 min each was carried out [3,16,20]. The sinusoidal loading was repeated for six cycles at 1-min period and continued for three cycles at 2-min period after a warm-up at a constant work load at the midpoint between maximum and minimum. On another day, another sinusoidal loading was repeated for three cycles at 5-min period and continued for two cycles at 10-min period after 5 min warm-up. Each sinusoidal period of oscillation was studied on a separate occasion (one session at a time, two sessions per week for each individual). The subjects walked on the treadmill at a freely chosen pace. The order of normoxic and hypoxic conditions was randomized, and the subjects performed one of these experimental measurements on one experimental day.

### Measurements

A mass-flow sensor (type AB; Minato Medical Sciences, Osaka, Japan) was attached to the expiratory port of the valve of the face mask worn by the subject to continuously record the subject’s expiratory airflow, which was calibrated before each measurement with a 3-L syringe at three different flow rates. We calculated the ${\dot{V}}_{E}$ values by integrating the tidal volume (VT; mL) and breathing frequency (B*f*; breaths·min^−1^) observed from flow tracings recorded at the subject’s mouth. We confirmed that the sensitivity of the hot wire was not changed with changes in the gas composition over the range of the physiological flow variations.

The end-tidal oxygen pressure (P_ET_O_2_; mmHg) and carbon dioxide pressure (P_ET_CO_2_; mmHg) were determined by mass spectrometry (WSMR-1400; Westron, Chiba, Japan) from a sample drawn continuously from the inside of the mouthpiece at 1 mL·sec^−1^. This ‘loss’ of gas volume was not examined in this study, because the loss of 1 mL·sec^−1^ was much smaller than the inspired and expired airflows [13,15]. Two reference gases of known composition (O_2_ 15.04%, CO_2_ 2.92%, and N_2_ 82.04%; O_2_ 11.93%, CO_2_ 6.96%, and N_2_ 81.11%) and room air were used to calibrate the mass spectrometer.

The volumes, flows, PCO_2_, and PO_2_ at the subject’s mouth were recorded in real time at 50 Hz sampling frequency by a computerized online breath-by-breath system (AE-280; Minato Medical Sciences) from time-aligned gas volume and concentration signals. Breath-by-breath ${\dot{V}}_{E}$ (BTPS), $\dot{V}O_{2}$ (STPD), $\dot{V}CO_{2}$ (STPD), VT, B*f*, P_ET_CO_2_, and P_ET_O_2_ were determined. The electrocardiogram (ECG), taken from a *V5*lead, was monitored continuously on an wireless ECG monitor (DS-2150; Fukudadenshi, Tokyo), and subject’s heart rate (HR) was measured by beat-by-beat counting of the R-spike of the ECG taken simultaneously with the other measurements. The signal controlling the speed of the motor driving the treadmill was delivered by a microcomputer through a digital-analog converter.

### Data analysis

All HR, ventilatory and gas exchange data were analyzed using a standard Fourier analysis as described previously [2,5,6,16,20]. In brief, the variation of the treadmill speed was regarded as the input function. The amplitude (*Amp*) and phase shift (*PS*; degree) of the fundamental component of the ${\dot{V}}_{E}$, $\dot{V}O_{2}$, $\dot{V}CO_{2}$, HR, VT, and B*f* were computed as follows:

$$Amp=({Re}^{2}+{Im}^{2}{)}^{0.5}$$ (1)

and

$$PS=\tan^{-1}\left(Re/Im\right)$$ (2)

where the *Re* and *Im* values are the real and imaginary parts of these responses determined after a second-by-second interpolation of these responses (*x*) as

$$Re=\frac{2}{NT}\sum_{t=0}^{NT}\left[(x(t)-Mx)\;cos(2\pi ft)\right]$$ (3)

$$\mathrm{and}\;Im=\frac{2}{NT}\sum_{t=0}^{NT}\left[(x(t)-Mx)\;sin(2\pi ft)\right]$$ (4)

where *x*(*t*) is the response value at time *t* (in seconds), *Mx* is the mean value of *x* for an integrated number of cycles (*N*), *T* is the period of the input signal (in seconds) and *f* (= 1/*T*) is its frequency. The *Amp* of each ${\dot{V}}_{E}$, $\dot{V}O_{2}$, $\dot{V}CO_{2}$, HR, VT, and B*f* parameter was normalized by dividing the ‘magnitude’ of each parameter obtained during steady-state walking at 3 and 6 km·h^−1^. We used the average data of the final 1 min of each period of steady-state walking to determine the ‘magnitude’. The *Amp* is presented as the *Amp* ratio (%) and the absolute values of the fundamental component of each variable.

### Statistical analysis

All values are presented as the mean ± SD. Significant differences in each variable (${\dot{V}}_{E}$, $\dot{V}O_{2}$, $\dot{V}CO_{2}$, VT, B*f*, HR, P_ET_CO_2_, and P_ET_O_2_) were determined by a two-way repeated measures analysis of variance (ANOVA) for comparing different FiO_2_ conditions (normoxia and hypoxia) × oscillation periods (*T*; 1, 2, 5, and 10 min). Tukey’s *post-hoc* test was applied for the appropriate data sets if a significant *F*- value was obtained. The statistical significance was set at p < 0.05.

## Results

**Fig 2:**
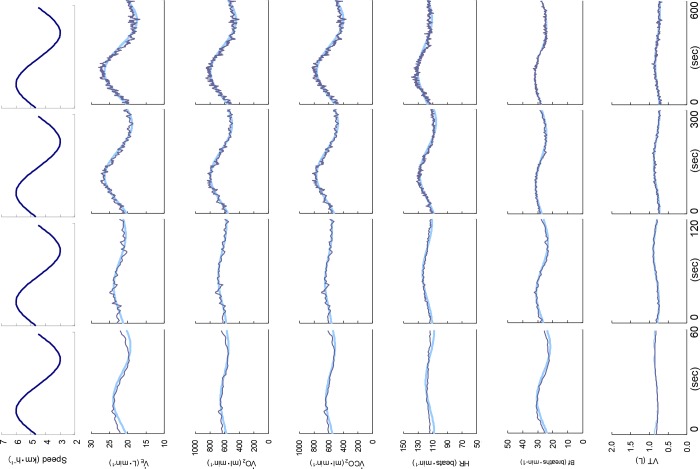
Time course of the ventilatory and gas exchange responses at different periods. The responses of a representative subject’s variables of ventilation (${\dot{V}}_{E}$), O_2_ uptake ($\dot{V}O_{2}$), CO_2_ output ($\dot{V}CO_{2}$), heart rate (HR), tidal volume (VT), and breath frequency (B*f*) over four different periods of 1, 2, 5, and 10 min are shown. Oscillating lines: Superimposed data on gas exchange variable. *Smooth lines*: Fundamental sine-wave component of these kinetics.

Although we did not measure the O_2_ saturation in each subject’s finger, the mean values of P_ET_O_2_ at the middle walking speed of 4.5 km·h^−1^ were 105.7 ± 2.8 mmHg under normoxia and 58.1 ± 9.4 mmHg under hypoxia. Fig 2 provides representative ${\dot{V}}_{E}$, $\dot{V}O_{2}$, $\dot{V}CO_{2}$, HR, VT, and B*f* kinetics data for one subject and the calculated fundamental components during sinusoidal walking for all periods. The *Amp* and *PS* were shown to be reliable variables for estimating the fundamental component of the ventilatory and gas exchange kinetics under hypoxia.

### Ventilatory and gas exchange kinetics between normoxia and hypoxia

**Fig 3:**
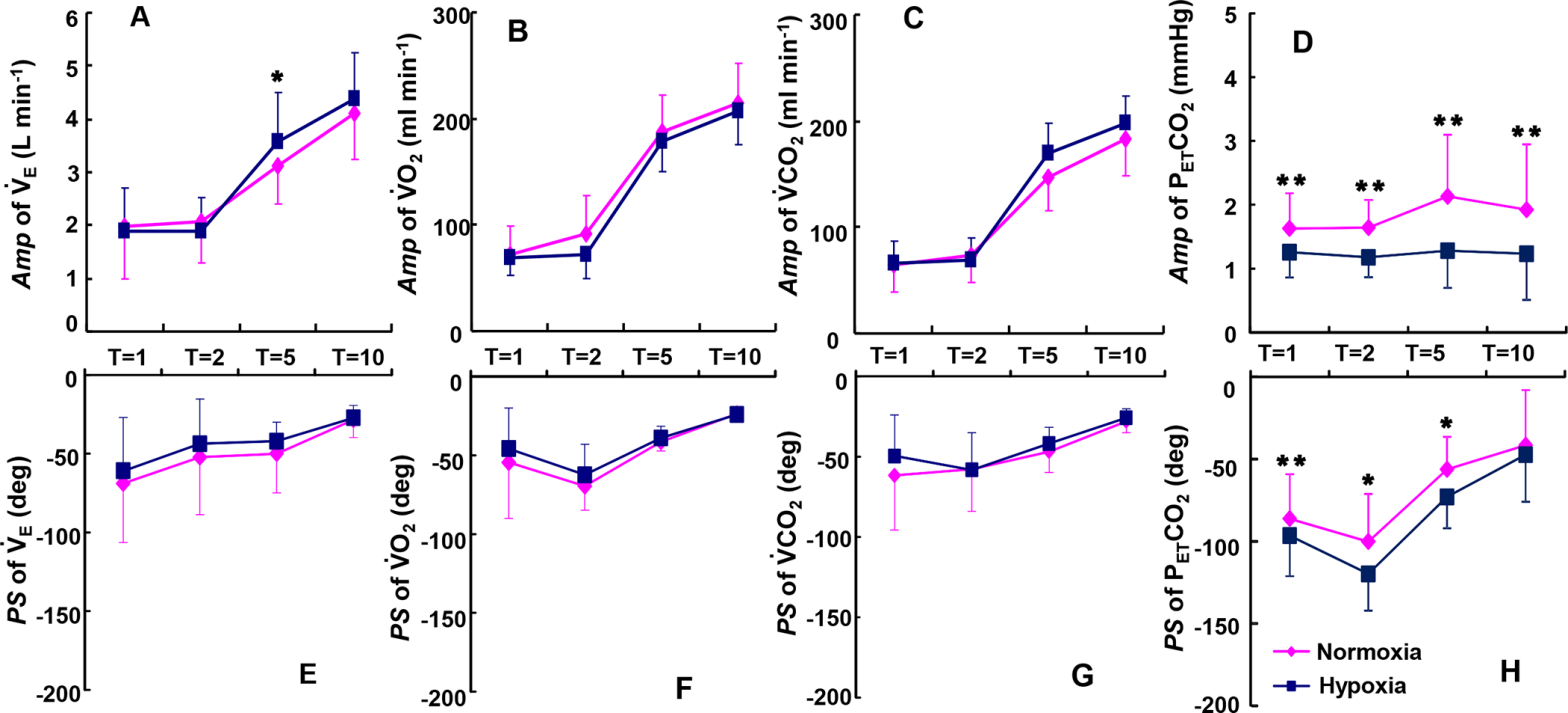
Comparison of *Amp* and *PS* of the kinetics between hypoxia and normoxia. Differences in the responses of *Amp* and *PS* of ${\dot{V}}_{E}$ (**A,E**), $\dot{V}O_{2}$ (**B,F**), $\dot{V}CO_{2}$ (**C,G**), and P_ET_CO_2_ (**D,H**) between hypoxia (blue line) and normoxia (red line) as a function of the periods of the sinusoidal changes in the treadmill speed are shown. *p < 0.05, **p < 0.01 for hypoxia vs. normoxia. Data are means ± SD.

During hypoxia, the *Amp* of the ${\dot{V}}_{E}$ response decreased from 4.39 ± 0.86 L·min^−1^ (during constant speed exercise) to 1.88 ± 0.83 L·min^−1^ as the period was shortened (Fig 3A). We observed a significantly greater *Amp* of the ${\dot{V}}_{E}$ only during the 5-min period during hypoxia compared to normoxia (p < 0.05). The *Amp* of the $\dot{V}O_{2}$ response during sinusoidal walking decreased from 207 ± 32 ml·min^−1^ (*T*: 10 min) to 70 ± 18 ml·min^−1^ (*T*: 1 min) under hypoxia.

There were no significant differences in the *Amp* of the VO_2_ in any of the periods between normoxia and hypoxia (Fig 3B). The *Amp* of $\dot{V}CO_{2}$ decreased from 197 ± 26 ml·min^−1^ (*T*: 10 min) to 67 ± 20 ml·min^−1^ (*T*: 1 min) without a significant difference between normoxia and hypoxia (Fig 3C). The changes in P_ET_CO_2_ during hypoxia displayed small *Amp* oscillations ranging from 1.2 ± 0.3 to1.2 ± 0.7 mmHg, and the *Amp* values at all periods became significantly smaller under hypoxia compared to those observed at normoxia (Fig 3D, p < 0.01).

The *PS* of all of the responses gradually increased at shorter periods during walking. The *PS* of the ${\dot{V}}_{E}$, $\dot{V}O_{2}$, and $\dot{V}CO_{2}$ responses were not significantly different between normoxia and hypoxia in any of the periods. Thus, the *PS* as an index of the time delay of ventilatory and gas exchange was not influenced by hypoxia (Fig 3E, 3F and 3G). The *PS* of the P_ET_CO_2_ response was significantly delayed between normoxia and hypoxia at all periods expect for 10 min (Fig 3H, p < 0.05).

### Hypoxic-induced slowing of the HR kinetics during sinusoidal walking

**Fig 4:**
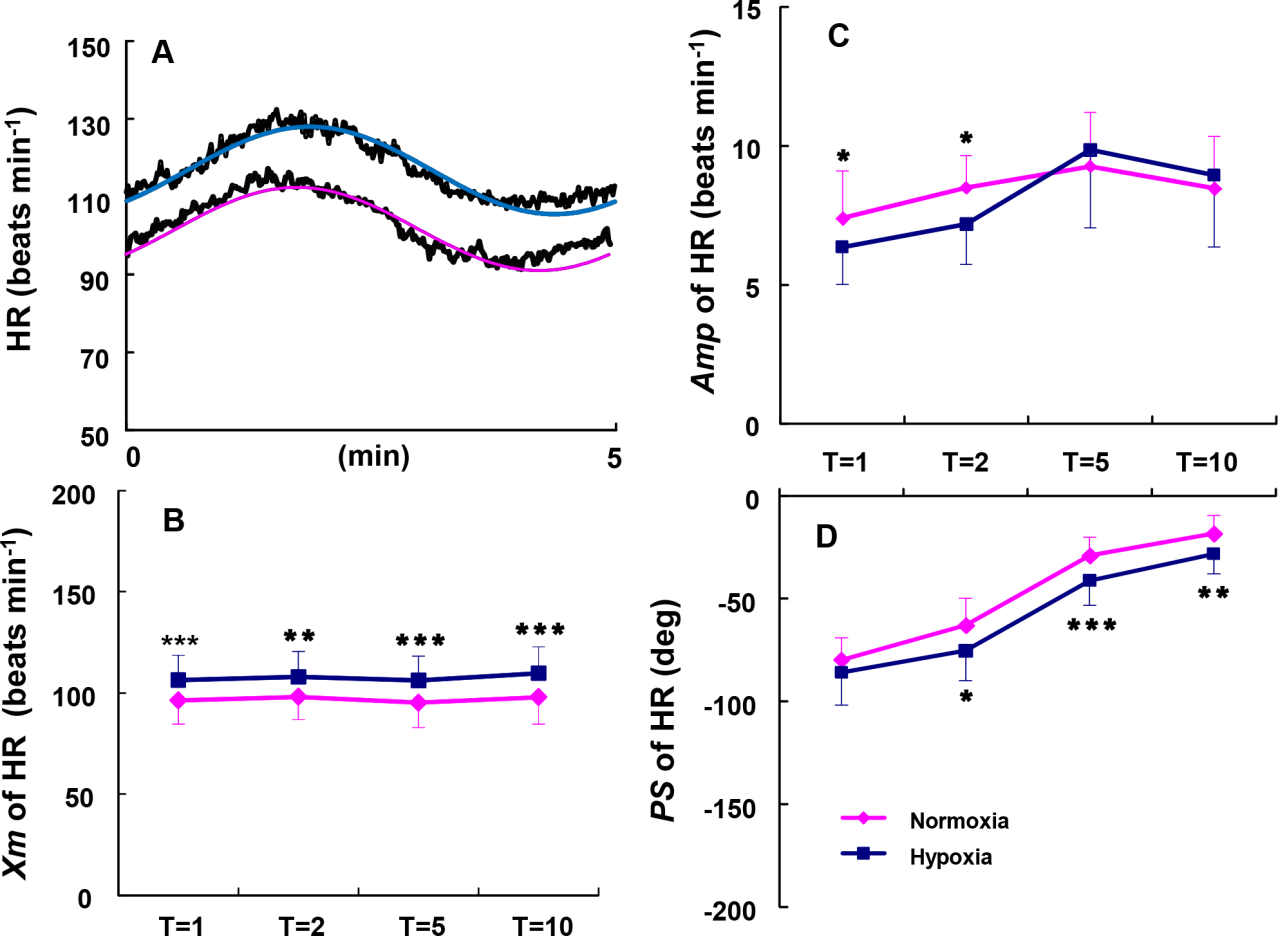
Comparison of the *Amp* and *PS* of HR kinetics between hypoxia and normoxia. Differences in the dynamic responses of a representative subject’s HR between normoxia and hypoxia at the representative sinusoidal period of 5 min are shown (**A**). The *Mx* (**B**), *Amp* (**C**), and *PS* (**D**) are shown for hypoxia (blue lines) and normoxia (red lines) as a function of the periods of the sinusoidal changes in the treadmill speed. *p < 0.05, **p < 0.01, ***p < 0.001 for hypoxia vs. normoxia. Data are means ± SD.

We compared the HR kinetics observed under hypoxia at the 5-min period with those observed under normoxia (Fig 4A). The average of the mean values (*Mx*) of HR was 97 bpm at all periods under normoxia, and the average of the *Mx* values of HR at all periods was significantly higher than approx.10 bpm at hypoxia (range: 106 and 110 bpm) (Fig 4B, p < 0.01, p < 0.001). The *Amp* of HR kinetics at the shorter periods of 1 min and 2 min were significantly smaller under hypoxia than normoxia (Fig 4C, p < 0.05), and the *PS* became significantly slower under hypoxia at the periods of 2, 5, and 10 min (but not the 1 min period) compared to the corresponding *PS* values under normoxia (Fig 4D, p < 0.05, p < 0.01, p < 0.001). Thus, hypoxia clearly induced an increase in *PS* (i.e., time lag) of the HR kinetics during sinusoidal walking.

### Changes in the mean values of ventilatory and gas exchange variables during sinusoidal walking

**Fig 5:**
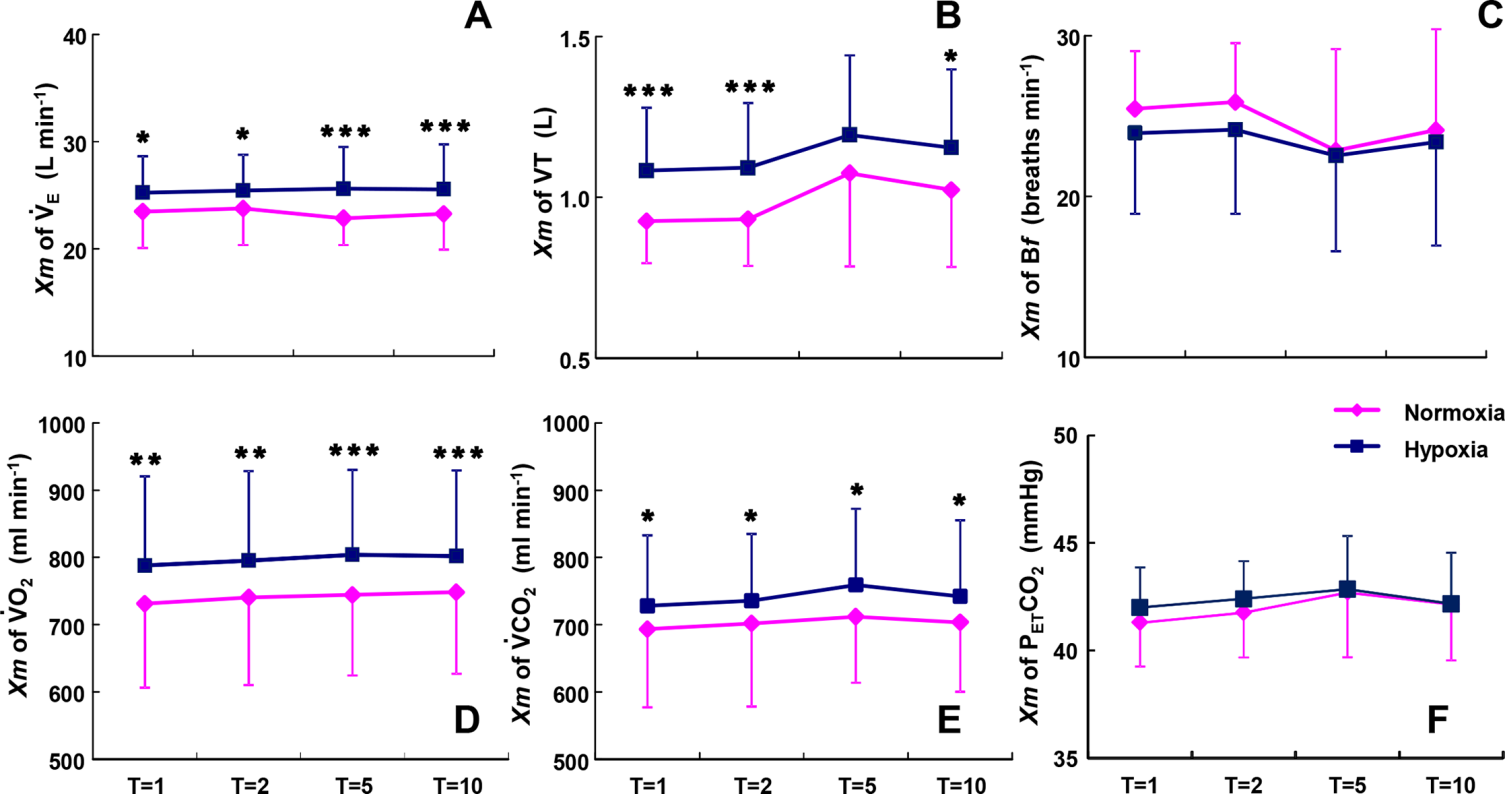
Comparison of the mean values of ventilatory and gas exchange kinetics between hypoxia and normoxia. Differences in *Mx* between hypoxia (blue line) and normoxia (red lines) are shown as a function of the periods of the sinusoidal changes in the treadmill speed. *p < 0.05, **p < 0.01, ***p < 0.001 for hypoxia vs. normoxia. Data are means ± SD.

The *Mx* values of the ${\dot{V}}_{E}$, $\dot{V}O_{2}$, and $\dot{V}CO_{2}$ kinetics were significantly different between hypoxia and normoxia (Fig 5A, 5D and 5E). The *Mx* of ${\dot{V}}_{E}$ was significantly elevated by 7.3% from 23.33 L·min^−1^ at normoxia to 25.47 L·min^−1^ under hypoxia (p < 0.05, p < 0.001). The *Mx* values of $\dot{V}O_{2}$ and $\dot{V}CO_{2}$ were also increased to 46 and 42 ml·min^−1^ (6.1%, 4.4%) under hypoxia compared to normoxia (p < 0.05, p < 0.01, p < 0.001) independently of the different periods. The *Mx* of VT became significantly greater under hypoxia compared to normoxia (p < 0.05, p < 0.001, Fig 5B), whereas the B*f* values were not significantly different between normoxia and hypoxia at any periods (Fig 5C). Thus, the greater *Mx* of ${\dot{V}}_{E}$ was attributable to the increased VT, not to B*f*. By contrast, the *Mx* of P_ET_CO_2_ was not significantly different between normoxia and hypoxia at each period, and it was narrowly distributed between 41.3 mmHg and 42.9 mmHg (Fig 5F).

### The ventilatory-gas exchange matching during sinusoidal walking

**Fig 6:**
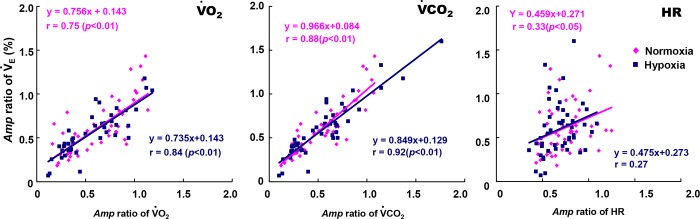
Relationship between the *Amp* ratio of gas exchange in hypoxia and normoxia. The *Amp* ratio of the ${\dot{V}}_{E}$ against $\dot{V}O_{2}$ (**A**), $\dot{V}CO_{2}$ (**B**), and HR (**C**) under conditions of either hypoxia or normoxia is shown. Note that the *A* ratio for the ${\dot{V}}_{E}$ kinetics followed the *Amp* ratio for the $\dot{V}CO_{2}$ kinetics more closely. In contrast, the *Amp* ratio for HR was not tightly related to the ${\dot{V}}_{E}$ kinetics. The regression line for the *Amp* ratio of the ${\dot{V}}_{E}-\dot{V}CO_{2}$ relationship was similar between hypoxia and normoxia: for hypoxia, y = 0.85x + 0.13, r = 0.92 (p < 0.01); for normoxia, y = 0.97x + 0.08, r = 0.88 (p < 0.01).

The *Amp* ratio (i.e., the *Amp* ratio for ${\dot{V}}_{E}$ between the constant and sinusoidal variation) of the ${\dot{V}}_{E}$ response was very closely related to the *Amp* ratio of $\dot{V}CO_{2}$ among the data obtained from all periods during hypoxia and normoxia (hypoxia, r = 0.92; normoxia, r = 0.88; p < 0.01) (Fig 6B). The slopes of the regression line for the ${\dot{V}}_{E}$ and $\dot{V}CO_{2}$ relationship were similar between hypoxia (0.849) and normoxia (0.966). A significant correlation between the *Amp* ratio of ${\dot{V}}_{E}$ and that of $\dot{V}O_{2}$ was also observed at both hypoxia (r = 0.84, p < 0.01) and normoxia (r = 0.75, p < 0.01) (Fig 6A). Moreover, the *Amp* ratio for ${\dot{V}}_{E}$ was significantly related to that of HR during normoxia (r = 0.33, p < 0.05), but not during hypoxia (r = 0.27, p = 0.73; Fig 6C).

## Discussion

Moderate hypoxia induced significant increases in the mean values of the HR, $\dot{V}O_{2}$, $\dot{V}CO_{2}$, and ${\dot{V}}_{E}$ kinetics, even though our frequency domain analysis of the $\dot{V}O_{2}$, $\dot{V}CO_{2}$, and ${\dot{V}}_{E}$ kinetics during sinusoidal walking revealed that the ventilatory and gas exchange kinetics under moderate hypoxia were similar to those under normoxia. In contrast, the kinetics of the HR response were slower under hypoxia compared to normoxia, and the HR kinetics were remarkably different from the ventilatory and gas exchange kinetics under moderate hypoxia. On the other hand, the slopes of the ${\dot{V}}_{E}$ and $\dot{V}CO_{2}$ relationship were not different between normoxia and hypoxia, which suggests that the ventilatory adjustment of hormonal drive (i.e., the CO_2_ drive) was equivalent between normoxia and hypoxia, even though hypoxic-induced hyperventilation (i.e., the *Mx* of ${\dot{V}}_{E}$) occurred via the peripheral chemoreflex during sinusoidal walking.

### Hypoxia-induced slowing of HR kinetics during sinusoidal walking

In the case of mild sinusoidal exercise such as slower walking (average HR ≤ 100 beats·min^−1^), the HR response will reflect mostly the parasympathetic nervous system [6,16]. Yamamoto *et al*. [21] demonstrated that cardiac vagal tone was greatly reduced beginning at >110 beats·min^−1^ in the HR response to incremental work; indeed, the average HR values under five different sinusoidal workloads ranged from 97 to 110 beats·min^−1^. Because hypoxia evokes sympathetic activity and lessens parasympathetic activity, the increase in the *Mx* of the HR from 100 bpm to 110 bpm in the present study reduced the parasympathetic activation under hypoxia.

The parasympathetic nervous activity appeared to have been reduced, judging from the greater *Mx* of the HR under hypoxia, and this seemed to account for the slower HR kinetics compared to normoxia. However, the HR declined to below 110 bpm at the minimum speed of 3 km·h^−1^, and thus we suspected that the parasympathetic activation would not completely disappear even under hypoxia. By contrast, as the averaged *Mx* of the HR under normoxia was approx. 100 bpm at any periods, the faster HR kinetics during walking would have occurred due to the alteration of predominant parasympathetic activation. Taken together, our findings demonstrate that the sinusoidal work loading used in the present study has an advantage in that the alteration of the midpoint work load between the minimum and maximum would modify the variation in the balance between sympathetic and parasympathetic outflow during exercise.

### Augmented mean values (*Mx*) of all variables by hypoxia

The *Mx* values of $\dot{V}O_{2}$ and $\dot{V}CO_{2}$ were significantly increased (6.1%, 4.4%) under moderate hypoxia compared to normoxia, as was also observed for the HR (8.8%). These observations provided evidence that non-steady-state exercise might induce an increase in energy expenditure without changing the kinetic characteristics of these variables. Despite this observation, it was unclear why sinusoidal exercise (i.e., non-steady-state exercise) was characterized by a significant increase in cardiac, ventilatory, and metabolic activities even under moderate hypoxic exposures. If patients with diseases such as heart failure or diabetes have limited walking speeds, hypoxia could help them achieve a higher energy expenditure, and a lower walking speed has also been suggested to be more protective of the muscles/joints in obese patients with orthopedic comorbidities than a higher walking speed [22].

Aaron *et al*. [23] reported that the energy expenditure for breathing (i.e., ${\dot{V}}_{E}$) amounted to approx. 2~3% of the total energy expenditure even during light exercise. Previous studies may have overestimated the cost of respiration during locomotion by ignoring the cost of increased HR [23]. However, the increased energy expenditure at severe hypoxia would be derived from the HR and ${\dot{V}}_{E}$ during walking; in our previous study using a bivariate regression model, the contribution of heart beats accounted for 60%~80% of the approx. 5% increase in energy expenditure during walking [24]. Our present finding of a deceleration in HR kinetics with an increase in the *Mx* suggests that hypoxia has a greater influence on the HR than on the ${\dot{V}}_{E}$ during sinusoidal walking. In other words, the energy expenditure of the heart beats may be somewhat greater than that of the breathing during walking under a hypoxic condition.

In another aspect of hypoxic hyperventilation, even though the *Mx* of ${\dot{V}}_{E}$ was significantly elevated by 7.3% from 23.33 L·min^−1^ at normoxia to 25.47 L·min^−1^ at hypoxia (p < 0.05, p < 0.001), the *Mx* of P_ET_CO_2_ was not significantly different between normoxia and hypoxia at each period. This observation indicated that hypoxia had induced a change in the chemoreflex drive [7–9], while the PCO_2_ was maintained at the same level under hypoxia or normoxia during walking at a sinusoidally changing speed. Although the physiological mechanism underlying this phenomenon was not clarified, sinusoidal exercise may provide a useful technique for the determination of the augmented energy expenditure and the increased ${\dot{V}}_{E}$ with the stable PaCO_2_.

### Ventilatory kinetics in response to exercise under a hypoxic condition

The carotid bodies are considered the primary mediators of kinetics at the transient phase in response to increases in the work rate [25]. It has been shown that under conditions of increased carotid-body gain induced by hypoxia, ${\dot{V}}_{E}$ kinetics are accelerated [7,8,26]. Conversely, reduced carotid-body gain in response to induced hyperoxia [7,8,26] or carotid-body resection [25] resulted in slowed ${\dot{V}}_{E}$ kinetics. In the present study, we did not observe faster ${\dot{V}}_{E}$ kinetics during sinusoidal walking even under moderate hypoxia, which suggests that the carotid-body gain in response to O_2_ is less affected by moderate hypoxia. At the same time, the $\dot{V}CO_{2}$ kinetics during walking showed a similar trend under normoxia and hypoxia, and thus it appears that hypoxic-induced hyperventilation can move the $\dot{V}CO_{2}$ response upward directly, but has less of an effect on the $\dot{V}CO_{2}$ kinetics under moderate hypoxia.

The increased ${\dot{V}}_{E}$ observed in the present study was attributed to the increased VT, not to B*f*. In their review regarding the effect of the interaction between VT and B*f* on the ventilatory response to various stresses, Tipton *et al*. [27] demonstrated that the increased ${\dot{V}}_{E}$ drive during moderate hypoxic exposure appears to be linked primarily to an increased VT in humans (see Fig 4). These findings may support the concept that the increased VT contribute more to the augmented ${\dot{V}}_{E}$ during sinusoidal walking under moderate hypoxia compared to B*f*.

Hypoxia would also induce less *Amp* and greater *PS* in the P_ET_CO_2_ response at all of the periods (using mathematical analysis) compared to those at normoxia. Consequently, the *Mx* values of the P_ET_CO_2_ were similar between normoxia and hypoxia at all periods. We considered that the *Amp* in P_ET_CO_2_ was less 1.3 mmHg, which was low enough to allow us to ignore the variation in P_ET_CO_2_ (i.e., the P_ET_CO_2_ response remained stable), and the *PS* of P_ET_CO_2_ was also meaningless from a physiological perspective. In other words, the alteration of P_ET_CO_2_ could reflect an error signal to the arterial and central chemoreceptors, which are further related to ${\dot{V}}_{E}$.

An important point needs to be clarified regarding PaCO_2_ homeostatics during hypoxic exercise. Previous investigations showed that during a sinusoidal change in walking speed, ventilatory-gas exchange matching occurred to prevent an abrupt increase in PaCO_2_, which would be derived from an increase in $\dot{V}CO_{2}$, thereby minimizing the changes in PaCO_2_ at an abrupt change in the exercise intensity [28–31]. This elaborate ventilatory system during mild exercise (i.e., walking) can be controlled under moderate hypoxia. However, in the cases in which more severe hypoxia is present during walking (e.g., at altitudes > 3,000m), it is not clear whether ventilatory and gas exchange matching will occur. In addition, the effective interaction between $\dot{V}CO_{2}$ and ${\dot{V}}_{E}$ kinetics may be supported by the findings we observed herein under hypoxic and normoxic conditions.

## Conclusion

In conclusion, moderate hypoxia could achieve an increase in the energy expenditure (i.e., increased $\dot{V}O_{2}$ and $\dot{V}CO_{2}$) and the increased ${\dot{V}}_{E}$ appeared to have occurred via the peripheral chemoreflex during non-steady-state walking at a sinusoidally changing speed. Our findings can advance the promotion of non-steady-state exercise protocols (e.g., sinusoidal walking) as available exercise for a greater energy demand even at lower exercise intensity in obese individuals and those with impaired movement. However, hypoxia produced the lesser changes in *Amp* and *PS* in ventilatory and gas exchange kinetics and the strong linkage between ${\dot{V}}_{E}$ and $\dot{V}CO_{2}$, which suggests that the ventilatory adjustment of hormonal drive (i.e., CO_2_ drive) was equivalent between normoxia and hypoxia. Our study has demonstrated that the ventilatory and gas exchange kinetics were remarkably different from a deceleration in HR kinetics under moderate hypoxia.

## Supporting information

S1 TableAmplitudes of gas exchange variables during sinusoidal walking under hypoxia and normoxia.Breath-by-breath ventilation (${\dot{V}}_{E}$, BTPS), O_2_ uptake ($\dot{V}O_{2}$, STPD), CO_2_ output ($\dot{V}CO_{2}$, STPD), and heart rate (HR) were determined. Data are shown by mean ± SD.(PDF)

S2 TablePhase shifts of gas exchange variables during sinusoidal walking under hypoxia and normoxia.Breath-by-breath ventilation (${\dot{V}}_{E}$, BTPS), O_2_ uptake ($\dot{V}O_{2}$, STPD), CO_2_ output ($\dot{V}CO_{2}$, STPD), and heart rate (HR) were determined. Data are shown by mean ± SD.(PDF)

S3 TableMean values of gas exchange variables during sinusoidal walking under hypoxia and normoxia.Breath-by-breath ventilation (${\dot{V}}_{E}$, BTPS), O_2_ uptake ($\dot{V}O_{2}$, STPD), CO_2_ output ($\dot{V}CO_{2}$, STPD), heart rate (HR), tidal volume (VT), breaths frequency (B*f*), and end-tidal PCO_2_ (P_ET_CO_2_) were determined. Data are shown by mean ± SD.(PDF)
