# Supplementary material for: Differential kinetics of the cardiac, ventilatory, and gas exchange variables during walking under moderate hypoxia
Source: PLoS One. 2018 Jul 25;13(7):e0200186. doi: 10.1371/journal.pone.0200186 (PMC6059434; doi:10.1371/journal.pone.0200186)
Supplement: S1 Table — Breath-by-breath ventilation (V˙E, BTPS), O2 uptake (V˙O2, STPD), CO2 output (V˙CO2, STPD), and heart rate (HR) were determined. Data are shown by mean ± SD. (PDF) [file pone.0200186.s001.pdf]

S1 Table. Amplitudes of gas exchange variables during sinusoidal walking under hypoxia and normoxia.

		V_E (L·min^{-1})	VO_2 (ml·min^{-1})	VCO_2 (ml·min^{-1})	HR (beat·min^{-1})
**Normoxia**	T = 1	2.0 ± 1.0	72.6 ± 26.7	64.4 ± 25.7	7.4 ± 1.7
	T = 2	2.1 ± 0.8	92.2 ± 35.7	74.0 ± 26.4	8.5 ± 1.2
	T = 5	3.1 ± 0.7	187.3 ± 34.9	147.1 ± 31.3	9.3 ± 1.9
	T = 10	4.1 ± 0.9	214.2 ± 37.4	183.2 ± 35.1	8.5 ± 1.9
**Hypoxia**	T = 1	1.9 ± 0.8	69.6 ± 17.7	66.7 ± 20.1	6.4 ± 1.3
	T = 2	1.9 ± 0.6	72.0 ± 22.2	69.7 ± 21.0	7.2 ± 1.5
	T = 5	3.6 ± 0.9	179.2 ± 29.6	170.1 ± 27.7	9.9 ± 2.8
	T = 10	4.4 ± 0.9	207.1 ± 31.8	197.5 ± 26.1	9.0 ± 2.6

Breath-by-breath ventilation (V_E, BTPS), O_2 uptake (VO_2 ,STPD), CO_2 output (VCO_2, STPD),and heart rate (HR) were determined. Data ae shown by mean±SD.
