# Supplementary material for: Differential kinetics of the cardiac, ventilatory, and gas exchange variables during walking under moderate hypoxia
Source: PLoS One. 2018 Jul 25;13(7):e0200186. doi: 10.1371/journal.pone.0200186 (PMC6059434; doi:10.1371/journal.pone.0200186)
Supplement: S2 Table — Breath-by-breath ventilation (V˙E, BTPS), O2 uptake (V˙O2, STPD), CO2 output (V˙CO2, STPD), and heart rate (HR) were determined. Data are shown by mean ± SD. (PDF) [file pone.0200186.s002.pdf]

S2 Table. Phase shifts of gas exchange variables during sinusoidal walking under hypoxia and normoxia.

		V_E (L·min^{-1})	VO_2 (ml·min^{-1})	VCO_2(ml·min^{-1})	HR (beat·min^{-1})
**Normoxia**	T=1	-68.8 ± 37.6	-54.6 ± 35.6	-61.7 ± 34.1	-79.8 ± 10.7
	T=2	-52.1 ± 36.6	-69.6 ± 15.3	-57.8 ± 26.3	-63.0 ± 13.2
	T=5	-50.0 ± 24.9	-41.4 ± 5.7	-46.8 ± 13.0	-28.9 ± 8.9
	T=10	-28.4 ± 11.3	-23.7 ± 3.0	-27.9 ± 7.3	-18.4 ± 8.9
**Hypoxia**	T=1	-61.0 ± 34.0	-45.8 ± 25.8	-49.7 ± 25.5	-85.9 ± 16.0
	T=2	-43.7 ± 28.2	-62.4 ± 19.4	-58.4 ± 23.2	-75.3 ± 14.7
	T=5	-42.1 ± 12.2	-39.1 ± 7.4	-41.9 ±10.0	-41.2 ± 12.1
	T=10	-27.3 ± 8.0	-24.2± 3.6	-25.9 ± 5.5	-28.2 ± 9.8

Breath-by-breath ventilation (V_E, BTPS), O_2 uptake (VO_2 ,STPD), CO_2 output (VCO_2, STPD),and heart rate (HR) were determined. Data ae shown by mean±SD.
