# Supplementary material for: Differential kinetics of the cardiac, ventilatory, and gas exchange variables during walking under moderate hypoxia
Source: PLoS One. 2018 Jul 25;13(7):e0200186. doi: 10.1371/journal.pone.0200186 (PMC6059434; doi:10.1371/journal.pone.0200186)
Supplement: S3 Table — Breath-by-breath ventilation (V˙E, BTPS), O2 uptake (V˙O2, STPD), CO2 output (V˙CO2, STPD), heart rate (HR), tidal volume (VT), breaths frequency (Bf), and end-tidal PCO2 (PETCO2) were determined. Data are shown by mean ± SD. (PDF) [file pone.0200186.s003.pdf]

S3 Table. Mean values (Mx) of gas exchange variables during sinusoidal walking under hypoxia and normoxia.

		V_E (L·min^{-1})	VO_2 (ml·min^{-1})	VCO_2(ml·min^{-1})	HR (beat·min^{-1})	VT (L)	B*f* (breaths·min^{-1})	$P_{ET}CO_2$ (mmHg)
**Normoxia**	T = 1	23.5 ± 3.4	731 ± 125	693 ± 116	96 ± 12	0.925 ± 0.131	25.4 ± 3.6	41.3 ± 2.0
	T = 2	23.8 ±3.4	741 ± 130	702 ±124	98 ± 11	0.931 ± 0.145	25.9 ± 3.7	41.8 ± 2.1
	T = 5	22.9 ± 2.5	744 ± 119	712 ± 98	95 ±12	1.075 ± 0.290	22.8 ± 6.3	42.7 ± 3.0
	T = 10	23.4 ± 3.3	748 ± 121	704 ± 103	98 ± 13	1.022 ± 0.239	24.1 ± 6.3	42.1 ± 2.6
**Hypoxia**	T = 1	25.3 ± 3.4	788 ± 133	728 ± 105	106 ± 12	1.083 ± 0.195	23.9 ± 5.0	42.0 ± 1.9
	T = 2	25.5 ± 3.3	795 ± 133	736 ± 100	108 ± 13	1.091 ± 0.201	24.2 ± 5.2	42.4 ± 1.8
	T = 5	25.6 ± 3.9	804 ± 126	759 ± 113	106 ± 12	1.194 ± 0.247	22.5 ± 6.0	42.9 ± 2.5
	T = 10	25.6 ± 4.2	802 ± 127	742 ± 113	110 ± 13	1.154 ± 0244	23.4 ± 6.5	42.2 ± 2.4

Breath-by-breath ventilation (V_E, BTPS), O_2 uptake (VO_2 ,STPD), CO_2 output (VCO_2, STPD), heart rate (HR), tidal volume (VT), breaths frequency (Bf),and end-tidal PCO_2 ($P_{ET}CO_2$) were determined. Data ae shown by mean±SD.
